# Magnesium production by molten salt electrolysis with liquid tin cathode and multiple effect distillation

**DOI:** 10.3389/fchem.2023.1192202

**Published:** 2023-07-03

**Authors:** Armaghan Ehsani Telgerafchi, Madison Rutherford, Gabriel Espinosa, Daniel McArthur, Nicholas Masse, Benjamin Perrin, Zujian Tang, Adam C. Powell

**Affiliations:** ^1^ Energy Metals Research Group, Department of Mechanical and Materials Engineering, Worcester Polytechnic Institute, Worcester, MA, United States; ^2^ Department of Chemistry, Cooperative Institute for Research in Environmental Sciences, University of Colorado Boulder, Boulder, CO, United States

**Keywords:** magnesium, molten salt electrolysis, distillation, reactive cathode, multiple effect thermal system, inert anode

## Abstract

Low-cost clean primary production of magnesium metal is important for its use in many applications, from light-weight structural components to energy technologies. This work describes new experiments and cost and emissions analysis for a magnesium metal production process. The process combines molten salt electrolysis of MgO using MgF₂-CaF₂ electrolyte and a reactive liquid tin cathode, with gravity-driven multiple effect thermal system (G-METS) distillation to separate out the magnesium product, and re-use of the tin. Electrolysis experiments with carbon anodes showed current yield above 90%, while a yttria-stabilized zirconia solid oxide membrane (SOM) anode experiment showed 84% current yield. G-METS distillation is an important component of the envisioned process. It can potentially lower costs and energy use considerably compared with conventional magnesium distillation. Techno-economic analysis including detailed mass and energy balances shows that this electrolyte composition could lower costs by utilizing CaO, which is the primary impurity in MgO, as the Hall-Héroult process uses the sodium impurity in alumina. Analysis options include: raw material types (magnesite rock vs. brine or seawater), drying and calcining using electricity vs. natural gas, and carbon vs. SOM anode type. Using SOM inert anodes results in a cost premium around 10%–15%, mostly due to higher electrical energy usage resulting from membrane resistance, and reduces GHG emissions by approximately 1 kg CO₂/kg Mg product. Capital and operating cost estimates, and cradle to gate greenhouse gas (GHG) emissions analysis under several raw material and process technology scenarios, show comparable costs and emissions to those of aluminum production.

## 1 Introduction

Magnesium metal (Mg) is well-known for its mechanical properties, which include exceptional specific stiffness, high specific strength, and mechanical damping capability. These favorable characteristics make it extremely advantageous in a wide number of industries, including transportation vehicles (Schumann, 2005; Skszek, 2015; Magnesium Vision, 2020; Powell et al, 2021). Its high oxidation energy and affinity for hydrogen make it also favorable for energy storage (Bergthorson, 2018; Röntzsch and Vogt, 2019). A significant barrier to greater use for these applications has been the lack of a Mg primary production technology with comparable cost, energy use, and environmental impact to those of the steel or aluminum which it would replace. Furthermore, the US and EU have both named Mg metal a critical material with very high supply risk and difficulty of substitution (European Commission, 2020; Federal Register, 2022), creating urgency for new production technologies.

Mg is commercially produced in two distinct ways: electrolysis of magnesium chloride (MgCl₂) or thermal reduction of magnesium oxide (MgO) using the Pidgeon process. Historically, electrolysis has accounted for nearly 75% of the global magnesium supply. This uses anhydrous magnesium chloride (MgCl₂) or carnallite (MgCl₂·KCl) feedstock made from brines, operates at temperatures ranging between 928 and 993 K, and produces liquid Mg metal and chlorine (Cl₂) gas at cathodes and anodes, respectively. This process results in life cycle cradle-to-gate greenhouse gas (GHG) emissions of 5–7 t carbon dioxide equivalent (CO₂e) per t Mg product (Ehrenberger, 2013; Xie et al, 2018; Ehrenberger, 2020), largely because of the expensive chloride drying operation, and handling of chlorine gas. A new variant at Alliance Magnesium uses a hydrogen anode to produce anhydrous HCl, and reduces the GHG impact to as low as 2.5 t CO₂e/t Mg (Fournier, 2021).

In the early 21st century, as China became the world’s largest producer of magnesium, the low cost of labor and thermal energy in China enabled the Pidgeon process to become dominant despite using more energy than electrolysis. Around 80 percent of the world’s primary magnesium metal is now produced in China via the Pidgeon method which is based on thermal reduction between 1373 and 1473 K with ferrosilicon as a reducing agent. The higher energy requirements, and use of coal and coke oven gas for thermal energy, result in GHG emissions usually around 20–27 t CO₂e/t Mg (Cherubini et al, 2008; Ehrenberger, 2013; Ehrenberger, 2020), with sulfur, soot and other emissions as well.

The introduction of the Hall-Héroult cell in the late 1800s dramatically reduced the cost of aluminum metal by using low cost aluminum oxide feedstock. A similar process produces most light rare earth metals such as neodymium. Unfortunately, two properties of magnesium prevent this process from reducing the cost and energy consumption of MgO reduction. First, Mg metal density is lower than all molten halide salts, so it would float and short-circuit the cell. Second, metallic Mg dissolves in many salts, resulting in electronic current and decreased efficiency.

To address both of these issues, Yerkes proposed a magnesium oxide electrolysis cell with a reactive cathode in the 1940s (Yerkes, 1947). The cathode of the cell is a dense liquid metal, such as tin, lead, copper or silver, which absorbs reduced magnesium metal to form an alloy with high density and low magnesium metal activity. Kang et al recently expanded on the concept using a MgF₂-LiF-MgO electrolyte with low melting point around 1023 K, low density, low volatility, and high conductivity, and measured current yield over 90% (Park et al, 2017; Kang et al, 2019; Lee et al, 2020; Lee et al, 2021a; Lee et al, 2021b; Jeoung et al, 2022; Kang et al, 2022). There are three important challenges with this electrolyte and process. First, MgO solubility is low, around 0.5–0.8 wt% at the 1,083–1123 K operating temperature range (Kim et al, 2020), compared with 8–15 wt% for alumina in cryolite (Zhang et al, 2003), such that maximum current density is likely low. Second, Ca and other electropositive raw material impurities accumulate in the electrolyte bath with no proposal for removing them. Third, Mg separation from the reactive cathode metal by vacuum distillation is a slow, costly, capital-intensive and energy-intensive batch process which consumes 5–7 MWh/t Mg product (Ditze and Scharf, 2008).

The variation on this process presented here would mitigate these issues by using MgF₂-CaF₂-MgO electrolyte with high MgO solubility (Krishnan et al, 2005; Krishnan, 2006), followed by gravity-driven multiple effect thermal system (G-METS) distillation (Powell, et.al, 2020; Telgerafchi et al, 2021; Rutherford et al, 2021; Espinosa et al, 2022; McArthur et al, 2023). G-METS can potentially reduce magnesium distillation energy consumption, capital cost, and other operating costs by as much as 90%. CaF₂ replaces LiF to inexpensively mitigate Ca accumulation in the electrolyte. MgF_2_ addition balances the CaO impurity via the *in situ* reaction: MgF₂ + CaO → MgO + CaF₂. This is analogous to AlF₃-NaF-Al₂O₃ electrolyte in the Hall-Héroult process with AlF₃ addition to balance Na₂O, which is the most prominent impurity in Bayer process alumina. This electrolyte also builds on years of research on MgF₂-CaF₂-MgO molten salt for MgO electrolysis using solid oxide membrane (SOM) anodes (Krishnan et al, 2005; Pal and Powell, 2007; DeLucas et al, 2008; Ditze and Scharf, 2008; Gratz et al, 2014; Guan et al, 2014) which place a yttria-stabilized zirconia (YSZ) solid electrolyte between the molten salt and anode. This research showed MgO solubility in MgF₂-CaF₂ eutectic is as high as 22 wt% (Krishnan et al, 2005), but maximum measured current yield reached just 67% (Pati, 2010). One drawback is the higher eutectic point of MgF₂-CaF₂, which is approximately 1,223 K, such that the electrolysis process must operate above 1,250 K, *cf.* MgF₂-LiF enables a lower process operating temperature of 1,053 K (Lee et al, 2021b).

The work presented here builds on a prior techno-economic analysis (TEA) of MgO electrolysis using this method by the authors (Rutherford et al, 2021). A design concept shows how a large electrolysis cell and potline could operate using YSZ SOM anodes, including using magnetohydrodynamics (MHD) for stirring. New experiments demonstrate molten salt electrolysis of MgO using a liquid tin (Sn) cathode in MgF₂-CaF₂-MgO electrolyte with both carbon and YSZ SOM anodes. These experiments show over 90% current yield using carbon anodes, and 84% current yield using YSZ SOM anodes, the latter of which is the highest Mg current yield to date with SOM anodes. And expansions on the prior TEA study include a detailed cost model of YSZ SOM anodes, and a cradle-to-gate GHG emissions estimate for the process.

## 2 Proposed magnesium production process

**FIGURE 1:**
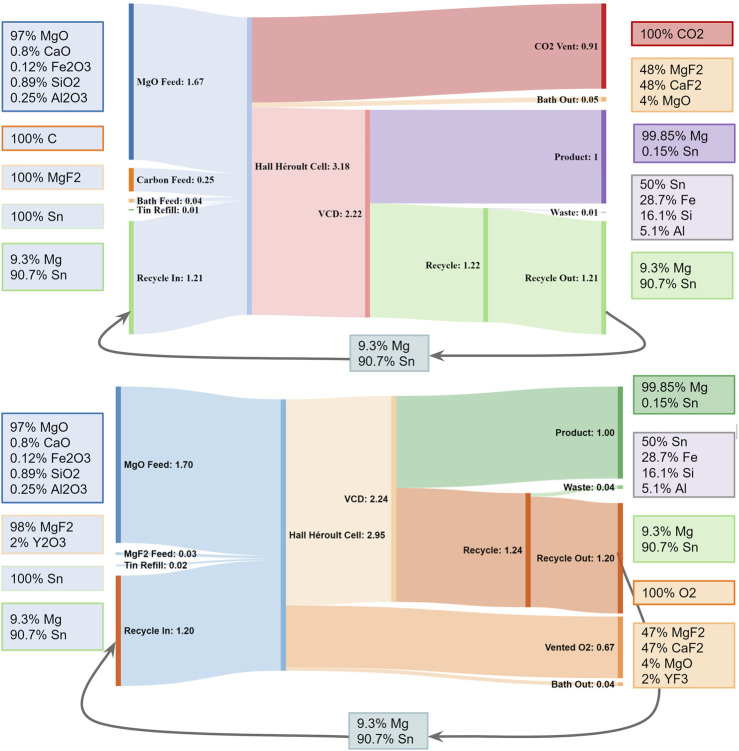
Combined flowsheet and Sankey diagram showing flow rates and example stream compositions for Mg production by the process described here. Using SOM anodes would replace the carbon anode feed with YSZ, with Zr and Y exiting via the “Waste” and “Bath Out” streams respectively, and “Vent” CO₂ would be O₂ instead. From (Rutherford et al., 2021).

The flowsheet and stream flow rates of the proposed Mg production process, using molten salt electrolysis with reactive cathode followed by G-METS distillation, are shown in Figure 1. The cathode begins as Sn-Mg alloy with 5–10 wt% Mg, or a similar alloy with lead. MgO reduction increases its volume to form between 60–40 and 66–33 Sn-Mg alloy, from which most of the Mg is later removed via G-METS distillation. The majority of the Sn is recovered post-distillation and circulated back into the electrolysis cell, with a small amount added to make up for losses.

Because the alloy in the distiller is mostly Sn, it cannot use the untreated steel alloys envisioned for Mg and rare earth magnet recycling, where everything in the distiller would be at least 50 wt% Mg. The distiller would thus need a liner material, such as electrodeposited molybdenum (Takeda et al, 2022) or a ceramic coating. Make-up Sn cost can be reduced by feeding tin oxide (SnO₂) in place of Sn, this would be preferentially reduced in the electrolysis cell over MgO to feed the cathode.

Other minor impurities in the MgO feed such as silicon, iron, and aluminum may build up in the liquid Sn, requiring occasional purging to keep their concentrations low and prevent them from interfering with process operation. One potential method for such purging could be cooling to a temperature close to the Sn melting point to reduce solubility of impurities and precipitate them out as a sludge.

Oxide ions from MgO react at the anode. Carbon anodes emit CO₂ while lowering the electrical energy requirement for the process due to the energetically favorable chemical reaction C + 2 O^2−^ → CO₂ + 4 e^−^. While YSZ SOM anodes provide the unique opportunity to prevent direct CO₂ emissions from the process, the oxygen evolution reaction and resistance of YSZ significantly increase the voltage and energy requirement of the process, as described in the prior TEA study (Rutherford et al, 2021). An anode design featuring a YSZ electrolyte coating on porous electronically-conducting perovskite anode, such as La_0.8_Ca_0.2_MnO_3-δ_, rare earth nickelates, or GDC-Pr_6_O_11_ (Nechache and Hody, 2021) could reduce the SOM thickness and cell resistance considerably. In either scenario, the cell would operate around 1,323 K, with 1 A/cm^2^ anode current density requiring cell voltage of about 5 V and 8 V for carbon and SOM anodes, respectively.

**FIGURE 2:**
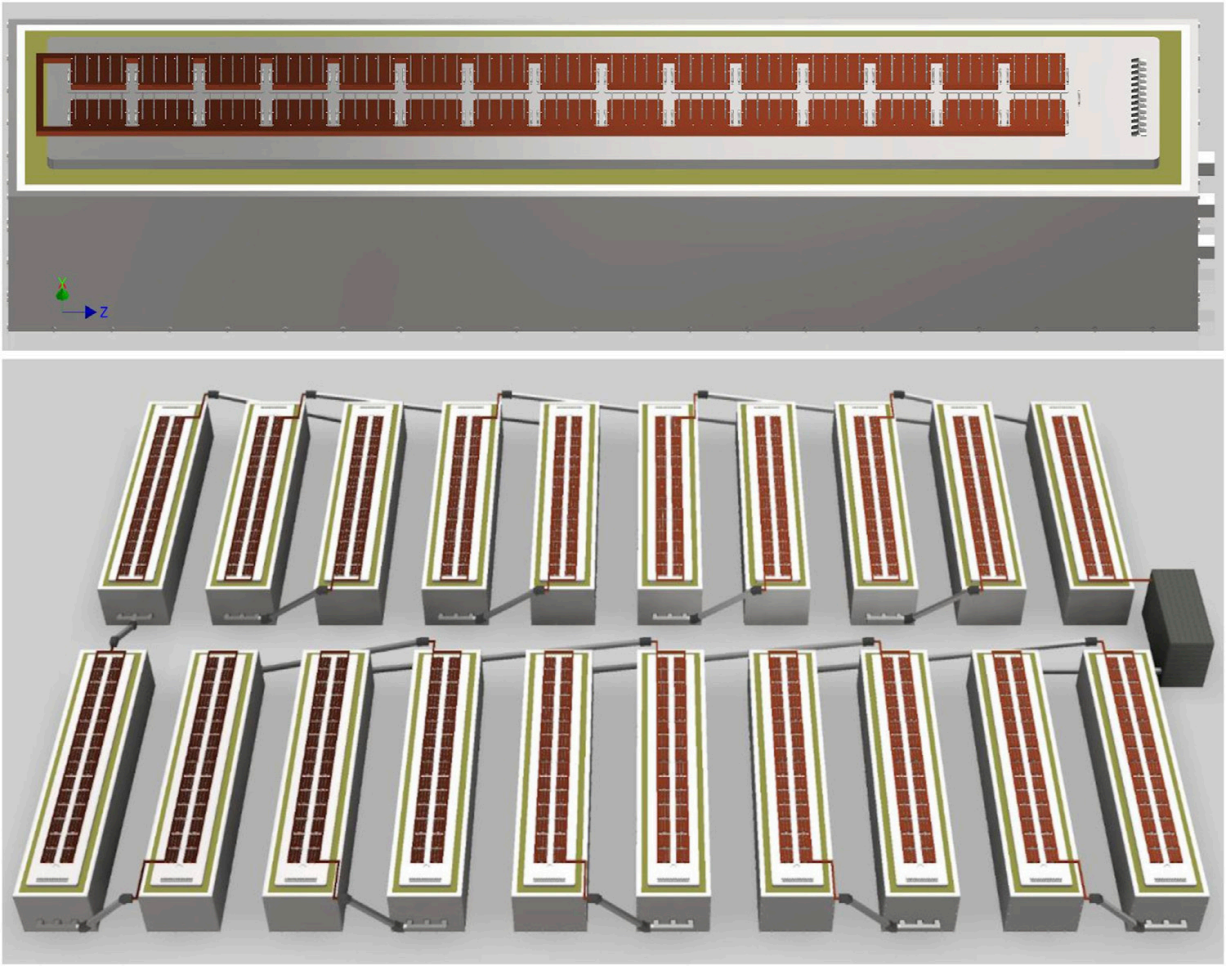
Concepts for a single cell (top) and small potline (bottom) using YSZ SOM anodes. The potline concept shows the rectifier at right.

A plant with carbon anodes would look very similar to a Hall-Héroult aluminum smelter, but one with YSZ SOM anodes would look quite different. Figure 2 shows a potential cell design, consisting of 30 clusters of 15 anodes each, for a total of 450 anodes, each carrying 400 A. These clusters can be switched off and removed independently to replace the anodes in the case of breakage or routine maintenance to address wear. The cathode uses steel rails joined by cast iron to graphite blocks similar to a Hall-Héroult cell. The cell and bus arrangement in Figure 2 would produce a mostly vertical magnetic field, which would interact with the radial component of current from each SOM tube to create vortices. This is important because the YSZ SOM cell lacks CO₂ gas lift stirring which drives mass transfer in cells with carbon anodes; MHD can instead provide that stirring.

## 3 Experimental procedures

Experiments characterized reduction of MgO from a 42 wt% MgF₂ 53 wt% CaF₂ 5% MgO (by mass) molten salt electrolyte with a reactive Sn cathode, forming a Mg-Sn alloy at the liquid tin cathode. Two experiments used a carbon anode, and one used a YSZ SOM anode with 3 wt% Y₂O₃ in the electrolyte to prevent yttria leaching from YSZ (Gratz et al, 2013).

**FIGURE 3:**
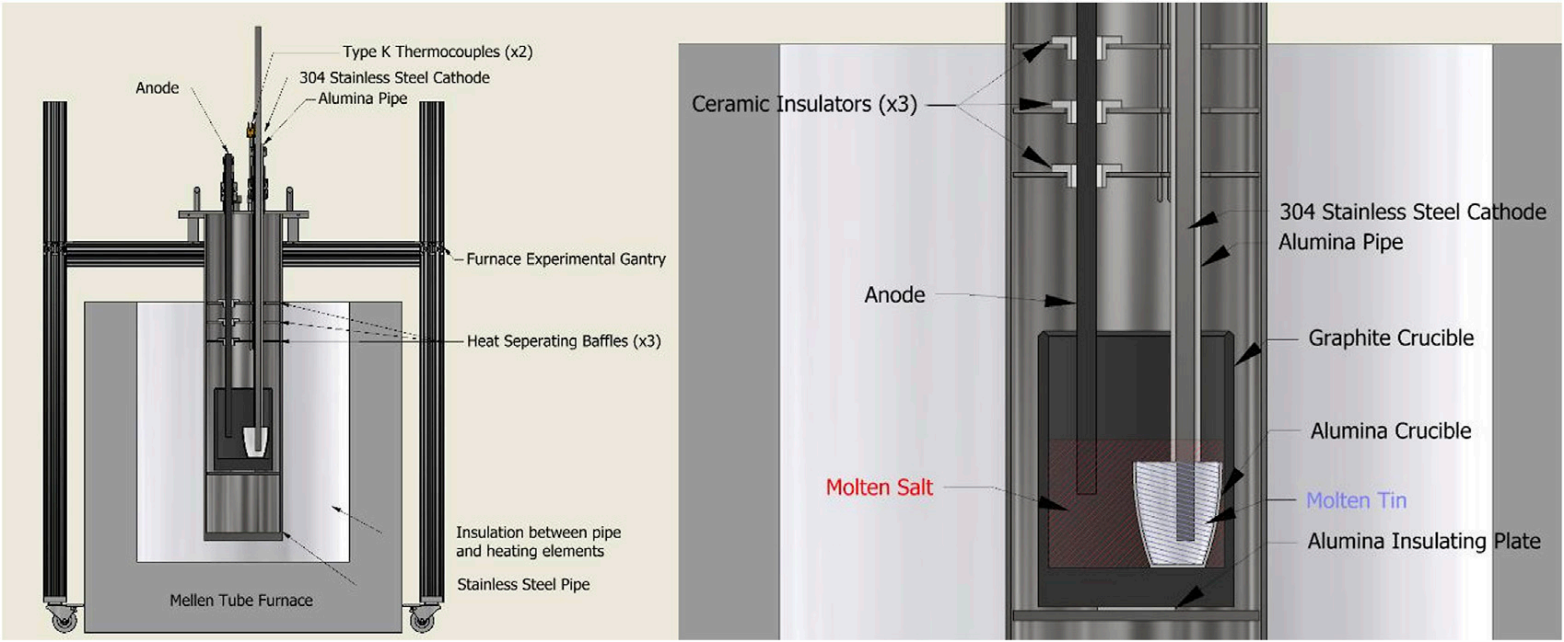
(Left) Diagram of the Mg production experimental setup using a carbon anode and steel cathode. (Right) Detailed view of “crucible cage” configuration within the furnace, making use of a nested crucible design.

Figure 3 shows the experimental apparatus used for electrolysis experiments. The experimental gantry and sealed furnace tube were made of aluminum and stainless steel respectively. To prevent oxidation of the graphite crucible or other components, argon gas entered the sealed furnace pipe at a rate of roughly 1 standard liter per minute (SLPM), displacing air and other gasses out through an exit tube. Exiting gases passed through an oxygen sensor before being vented away from the experiment. When oxygen content fell below 0.1%, heating and electrolysis could begin.

Carbon anodes were 0.75″ diameter graphite rods (graphitestore.com fine extruded rod). The YSZ SOM anode consisted of a 0.75″ outer diameter (OD) 2–3 mm thickness closed-end YSZ solid electrolyte tube, with 5–10 g silver inside acting as the anode, and 0.25″ diameter graphite rod current collector for simplicity and robustness.

Salts were first baked in a box furnace at 300°C for 4 h to prevent any adverse water reactions due to bath salt or oxide hydration in ambient air. In powder form, the various salts have low densities, so they were blended and melted together in a graphite crucible at 1,150°C for 12 h to reduce their solid volume by more than 50%. After cooling, the solidified salt block was extracted from the crucible and broken apart using a rock crusher to <2 cm pieces. This mixture was much more dense than its previously powdered form, making room for the other 1/3 total experimental mass of salts within the crucible. In cases with smaller salt quantities, the entire prebaked mass of salts fit within the crucible to be pre-melted.

To prevent current leakage from the molten salt reaction throughout the apparatus framing, various techniques of electrical insulation were used. As seen in Figure 3, the crucible cage used 3 ceramic bushings to prevent contact between the anode and stainless steel heat separating baffles. An alumina pipe separated the stainless steel cathode current collector from the baffles as well as the molten salt. The graphite crucible was placed atop an alumina plate and wrapped in ceramic fiber insulation to prevent current leakage to the body of the cage assembly. Similar precautions were taken outside the furnace; atop the lid assembly, various PTFE bushings are used to insulate the electrodes from the couplings which hold them in place.

For two of the experiments, a secondary alumina crucible separated the liquid Sn-Mg cathode from the graphite crucible, following Lee et al. (2020). (In the first carbon anode experiment the tin was in the bottom of the main graphite crucible.) Solid tin shot or lump (99.9+% pure, MetalShipper.com) was added to the alumina crucible before setting it within the larger graphite crucible. This nested crucible design meant the Sn-Mg cathode, when molten, was contained within the alumina crucible, only contacting the molten salt at the top interface of the crucible; the stainless steel cathode current collector also did not have electrical connection with the molten salt except via the Sn-Mg cathode. Once set, the alumina pipe was lowered and secured flush with the rim of the alumina crucible, the steel cathode was then lowered through the pipe until it rested atop the solid tin shot. The anode was then also lowered into position, a few centimeters from the bottom of the graphite crucible. After the electrodes were in place, the crushed pre-melted salt was added to the graphite crucible, concealing the alumina crucible entirely. Finally, any remaining prebaked salt mixture was added to the larger crucible before setting and sealing the crucible cage into the furnace pipe apparatus.

Because these experiments resulted in relatively low Mg concentration in Sn, a first “experiment zero” measured the concentration of Mg picked up by Sn during static exposure to the molten salt bath, without electrolysis. This experiment began with the following ingredients in a graphite crucible: 48.8 g tin lump (99.8% Strem) with a blend of powders consisting of 30.5 g MgF₂ (42.1 wt%), 38.3 g CaF₂ (52.8 wt%) and 3.7 g MgO (5.1 wt%). The crucible and its contents were heated in argon gas in stages to 1,050°C as follows: ramp to 500°C at 10°C/min, hold for 2 hours, ramp to 800°C at 10°C/min, hold for 30 min, and then ramp to 1,050°C at 10°C/min. This condition was held for 30 min, then the furnace power was shut off and the system cooled. The ramp-hold procedure was intended to drive off any moisture as water vapor; if ramped rapidly to 1,050°C, some of it might have reacted with fluoride salts to form HF.

All electrolysis experiments used this same heat-up procedure, though the one with a YSZ SOM anode used a slower heating rate of 2°C/min for all ramps. These ramp and set temperatures were controlled at the furnace thermocouple, which was often 20°C–50°C above the measured temperature inside the crucible.

### 3.1 Electrolysis procedure

**FIGURE 4:**
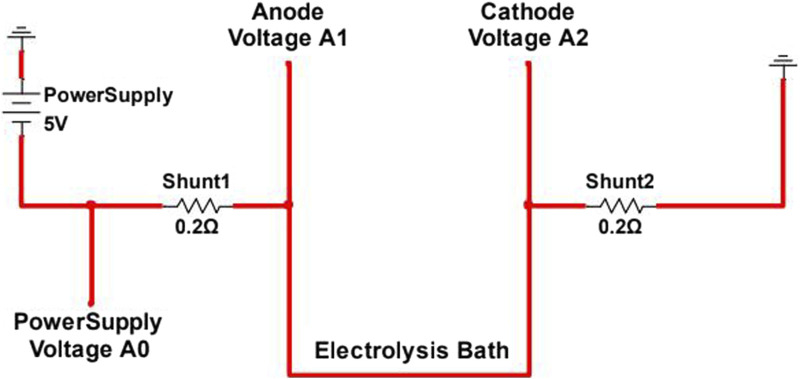
Electrolysis wiring diagram for electrically isolated experimental electrolytic magnesium production cell.

Once the temperature measured inside the crucible stabilized at 1,050°C, the electrodes were lowered to 0.5–1 cm from the bottom of the crucible. Power supply leads were connected to the electrodes with 0.02Ω shunt resistors in the lines to measure current in each electrode, as shown in Figure 4. The anode was then raised 1 cm and the power supply turned on. A voltage sweep was run, starting at open circuit voltage (OCV) and increasing by 0.5 V/min to 5 V, after which the current was stopped and voltage returned to OCV. The voltage was then held at 1 V for 30 min in order to remove more electronegative impurities present in the bath. Then after another sweep from OCV to 5V, voltage was held at 3 V for 60 min. Another sweep was performed, and the voltage was held at 3V for another 60 min. A final sweep was performed before the furnace was shut down and the products allowed to cool for 12 h.

A custom-made data logger, using an Arduino Mega, measured the voltage at points A0, A1, and A2, as shown in Figure 4. The resulting voltages, in combination with the known shunt resistances, were used to calculate the cell current at various points in the electrolysis process.

After completing the electrolysis process, electrodes were disconnected from the power supply, and the furnace was turned off, allowing the molten salts and metals to solidify and cool for 12 h. The argon gas flow was left running during the cooldown process to prevent potential component oxidation.

Each electrolysis experiment involved multiple voltage sweeps, generally from 0 to 5 V, and potentiostatic holds, generally at 2 V or 4 V. Electrolysis current measured by I=(VA1-VA0)/0.2Ω was integrated over time, and total charge provided an estimate for magnesium metal reduction mass *m̅*_Mg_ based on the expression:

$${\bar{m}}_{Mg}=\frac{M_{Mg}\int I\;dt}{2F}$$

where *M*_Mg_ is the molar mass of magnesium, *I* is current, and *F* is Faraday’s constant. The current yield is then the ratio of actual mass to estimated mass *m*_Mg_ ÷ *m̅*_Mg_ where *m*_Mg_ is the product of measured mass of Mg-Sn alloy and its adjusted mass fraction measured by ICP-OES:

$$m_{Mg}=m_{MgSn}(y_{Mg}-y_{Mg}^{\prime })$$

where *y’*_Mg_ is the mass fraction of Mg in Sn-Mg alloy resulting from contact with the bath (experiment zero).

### 3.2 Sample collection and characterization

After electrolysis was complete and the furnace has cooled to room temperature, the experimental cage was removed from the furnace pipe. Upon removing and turning over the graphite crucible, the solidified salt, containing the alumina crucible, slid out of the large crucible. The solid salt “puck” was then broken apart using a hammer, destroying the alumina crucible in the process. Once broken apart, the solid MgSn alloy, shaped to the volume of the alumina crucible, was extracted.

Sample composition analysis used Inductively Coupled Plasma–Optical Emission Spectrometry (ICP-OES, Perkin Elmer Optima 8,000). Because the MgSn alloy did not have a uniform composition, a hacksaw created a vertical cross-section by cutting the alloy from top to bottom, in order to get a representative average composition. The shavings of this cut were collected and dissolved for ICP-OES.

### 3.3 Cradle-to-gate emissions estimation

Estimating GHG emissions per tonne of Mg production begins with defining the scope of analysis. This work is a partial cradle-to-gate analysis, that is, it includes emissions due to MgO raw material production, carbon or YSZ SOM anode production, and carbon anode consumption in electrolysis. But it does not include dolomite or magnesite mining, nor transportation, nor minor additions such as tin (or tin oxide) and MgF₂. This is therefore not a complete life cycle analysis (LCA) study. There are multiple variables including raw material production options, and anode type. This analysis focuses on the following three scenarios.

• Low emissions: MgO from brine/seawater precipitated by chlor-alkali NaOH; ultrasonically dried and calcined using electrical resistance heating; reduction using SOM anodes.
• Medium emissions: MgO from brine/seawater precipitated by lime/dolime; dried and calcined using natural gas; reduction using carbon anodes.
• High emissions: MgO from magnesite calcined using natural gas; reduction using carbon anodes.

The model assumes drying and calcining operations are 50% efficient, that is, they consume twice as much natural gas or electrical energy as the heat of vaporization for water and enthalpy of the reaction Mg(OH)₂ → MgO + H₂O or MgCO₃ → MgO + CO₂. It assumes electricity comes from hydropower at the IPCC median estimate of 24 gCO₂e/kWh (Schlömer et al, 2014).

## 4 Results

In Sn-bath exposure (experiment zero), Mg:Sn mass ratio in the metal phase following exposure was measured using ICP-OES and reported as 0.0001050, 0.0001229 and 0.0001501. This indicated that the Sn gained approximately 0.0126 wt% Mg from contact with the bath; this is the value of *y’*_Mg_ in Eq. 2.

### 4.1 Electrolysis experimental results

Table 1 summarizes the results of electrolysis experiments. Experiments 1 and 2, which used carbon anodes, showed the highest current yields at around 94% and 97% respectively, with the YSZ SOM anode experiment showing relatively high current yield above 84%, as mentioned above.

**TABLE 1:** Summary of results from electrolysis experiments.

Experiment	Anode	*∫ I dt*, A·h	Est. *m̅*_Mg_, g	Measured *y*_Mg_ (%)	Result *m*_Mg_, g	Current yield (%)
1	C	5.68	2.57	0.264	2.51	97.5
2	C	0.692	0.313	0.084	0.294	93.8
3	SOM	7.43	3.36	1.476	2.847	84.7

**FIGURE 5:**
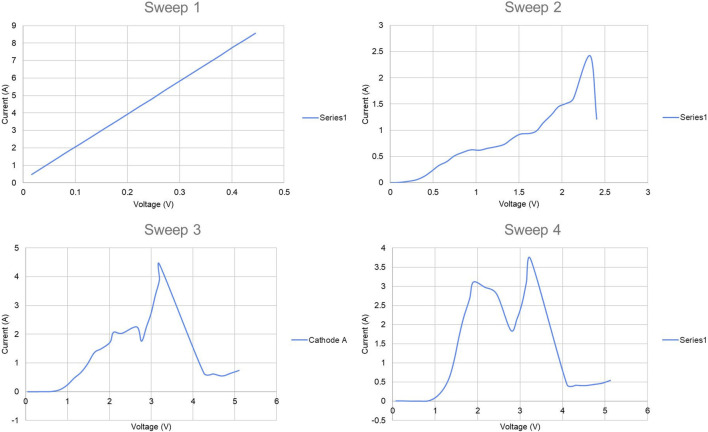
Current vs. voltage in potentiodynamic sweeps of electrolysis experiment 1.

**FIGURE 6:**
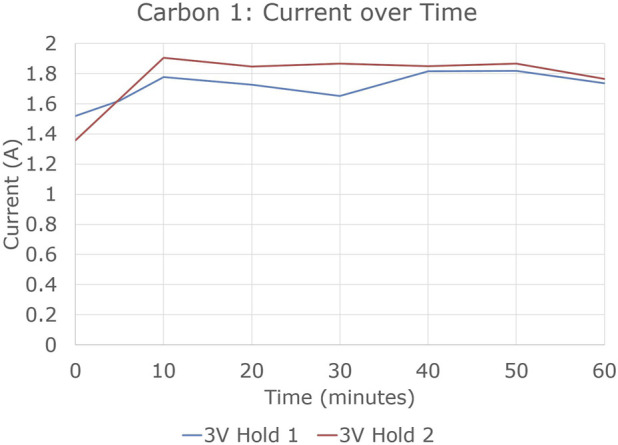
Current vs. time in potentiostatic holds of electrolysis experiment 1.

Figure 5 shows the current-voltage relationships in the four potentiodynamic sweeps of Experiment 1, all of which increased voltage at 0.5 V/min. Figure 6 shows current vs. time in its potentiostatic holds. The overall experiment ran as follows.

• Sweep 1: cut short as its linear shape indicated a short circuit of the anode to the liquid tin cathode which covered the bottom of the crucible; raised the cathode current collector 1 cm.
• Sweep 2, 0.332 A·h charge: nonlinear I-V curve indicated electrolysis, but significant current below 1 V indicated presence of impurities more electronegative than Mg (*e.g.*, Fe, Si, Cr).
• Potentiostatic hold at 0.75 V, 0.0006 A·h.
• Sweep 3, 0.803 A·h: very low current below 1 V indicated reduced impurity content.
• Potentiostatic hold at 3 V, 1.734 A·h.
• Sweep 4, 0.994 A·h: again low current below 1 V.
• Potentiostatic hold at 3 V, 1.816 A·h.

At 1,050°C, the free energy *ΔG* of the reaction 2MgO + C → 2 Mg + CO₂ is 478 kJ/mol at unit activities and 1 atm (Linstrom, 1997), so reference potential is −*ΔG/nF* = −1.24 V where *n* is the number of electrons transferred (in this case four) and *F* is the Faraday constant. This corresponds roughly to the voltage axis intercept of a tangent line to the main current-voltage trend between 1 and 2 V, indicating this is likely the primary reaction taking place. Both the MgO reactant and Mg product are dissolved, the former in the electrolyte and the latter in tin, so these reduced activities should not significantly affect reference potential. Note that when applied voltage is above about 2–2.5 V, current drops. This is likely due to “anode effect” at the carbon anode, a phenomenon involving perfluorocarbon generation and anode surface passivation.

**FIGURE 7:**
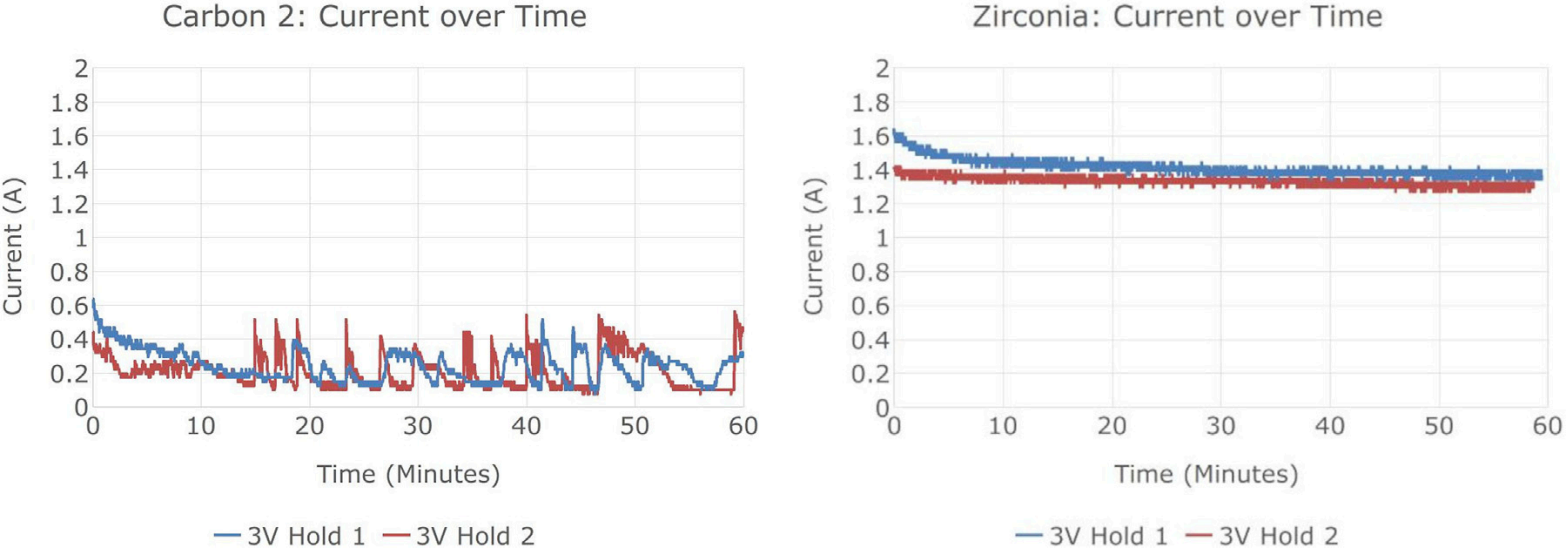
Current vs. time in 3 V potentiostatic holds of electrolysis experiments 2 and 3.

Experiments 2 and 3 were similar to experiment 1. Their potentiostatic hold currents are shown in Figure 7. Experiments 1 and 3 showed much higher current, and much more steady current, than experiment 2.

**FIGURE 8:**
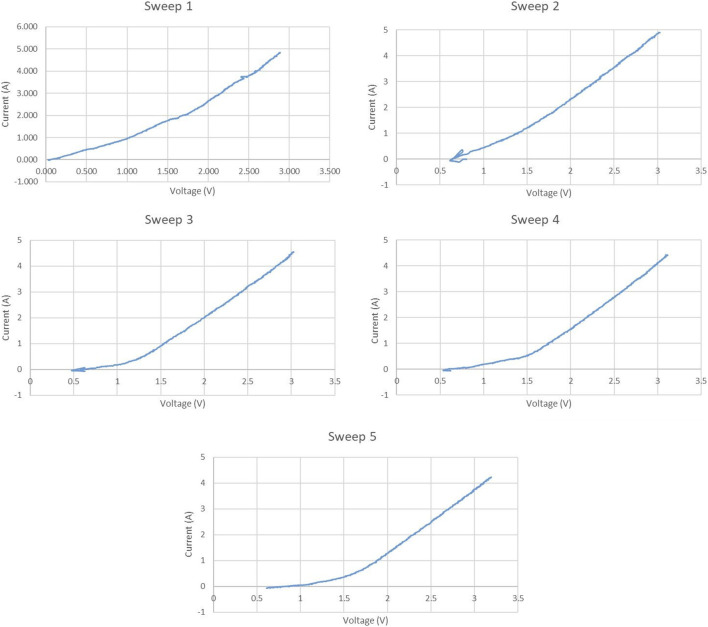
Potentiodynamic sweeps in electrolysis experiment 3.

Figure 8 shows the potentiodynamic sweeps of experiment 3, again at 0.5 V/min. A fifth sweep was added at the end of the experiment. The graphite current collector in the SOM anode resulted in the same overall reaction of 2MgO + C → 2 Mg + CO₂, instead of 2 MgO → 2 Mg + O₂ which would have resulted from inert current collector use. tangent voltage axis intercept of 1.1–1.4 V, corresponding roughly to the −1.24 V potential of the reaction 2MgO + C → 2 Mg + CO₂. The increase of that intercept over time from Sweep 1 to Sweep 5 likely indicated progressive removal of more electronegative impurities than Mg from the electrolyte.

### 4.2 Techno-economic analysis

An initial techno-economic analysis (TEA) describing the industrial-scale process was previously described in Rutherford et al (2021) for a 20,000 ton/year plant consisting of a potline of 46 electrolysis cells at 180 kA. That TEA consisted of full material and energy balances for the magnesium electrolysis process, balancing 16 elements with the ability to adjust for different feedstock and product compositions. The resulting operating cost estimate for production using carbon anodes was $2,060–3,690/t, with capital cost estimate of $11,000 per t/a.

**FIGURE 9:**
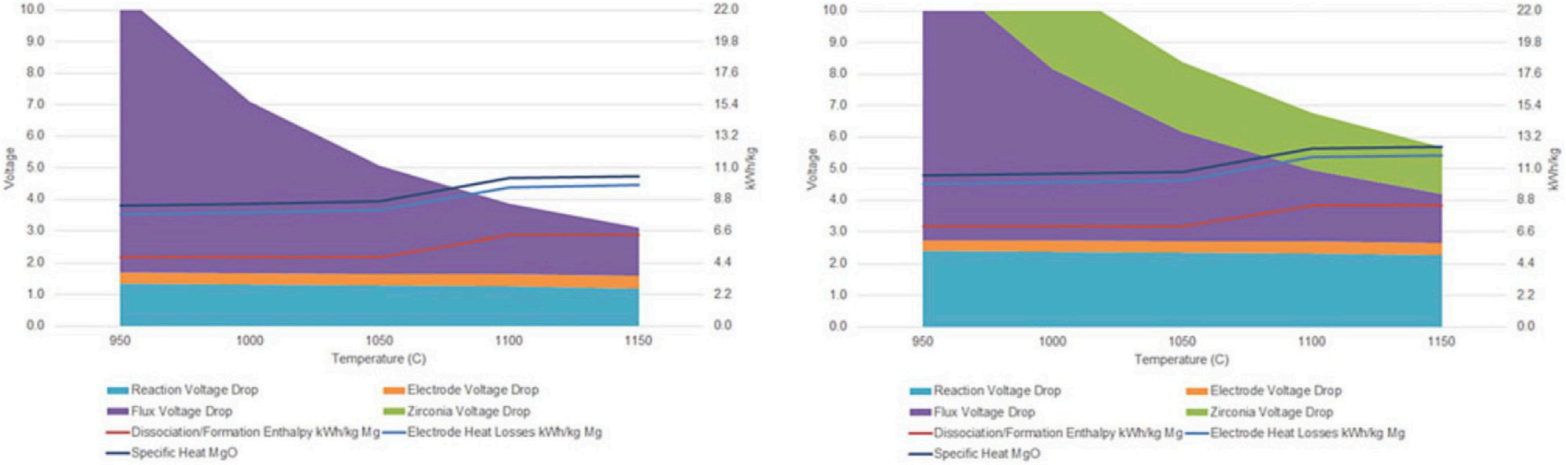
Electrical and electrochemical overpotentials (solid stacked colors) as functions of temperature using carbon anodes (left) and YSZ SOM anodes (right), showing corresponding thermal energy use (stacked line graph). From (Rutherford et al., 2021).

Figure 9 shows cell voltage and energy use as a function of temperature at fixed current density and anode-cathode distance for carbon and SOM anodes. At higher temperature, resistance of the electrolyte flux and YSZ SOM both decrease, as does reaction free energy and potential. But enthalpy of the reduction reaction increases above the Mg boiling point at 1,090°C. The excess shaded stack height above stacked line height indicates that resistance heating in the electrolyte is sufficient to provide enthalpy of the endothermic reaction, sensible heat of MgO feedstock, and minimum electrode heat losses for temperature up to about 1,090°C with carbon anodes and 1,150°C with SOM anodes. (Operating above the boiling point is currently not envisioned.)

This work expands the TEA to include detailed costs of YSZ SOM anode production instead of carbon anodes. It is assumed that across the 46 operating electrolysis cells, each cell will have 450 zirconia tubes with 40 cm bath immersion depth (50 cm total length), 3.6 cm inner diameter and 4.0 cm outer diameter, for total tube mass of 0.73 kg. This immersed area leads to 0.9 A/cm^2^ average current density at the SOM tube interior, somewhat less at the outer surface. With estimated lifespan of 4,000 h, just under 50,000 SOM tubes would be consumed each year. This lifespan is based on static measurements of YSZ erosion rate over up to 500 h in MgF₂-CaF₂-MgO bath with Y³⁺ added to the bath to minimize yttria leaching from the YSZ (Gratz et al, 2013).

The zirconia production process consists of five steps: milling YSZ powder, mixing with binder and other ingredients, extrusion, firing, and testing. Material is extruded into a tube shape with one end formed into a hemispherical cap. The modeled costs for each operation are given in Table 2, and given the tube production rate above with 90% powder utilization and other losses below, YSZ consumption would be just over 51,000 kg/a. Assuming a YSZ price of $50/kg at scale, and total annual manufacturing cost of $853 k, total tube production cost is $3.96M/year, or $198/t Mg product, of which more nearly 70% is spent on the YSZ raw material.

**TABLE 2:** Detailed cost estimates for YSZ production.

	Units	Milling	Mixing	Extrusion	Firing	Testing/QA
Zirconia powder or parts entering	(kg or parts)/year	54,436	54,436	63,991	58,871	54,750
Yield	%	100%	95%	92%	93%	90%
Powder or parts exiting	(kg or parts)/year	54,436	51,714	58,871	54,750	49,275
Machine total cost	$ capital	$ -	$80,000	$2,000,000	$1,000,000	$100,000
Depreciation lifetime	years	7	5	7	5	6
Machine cost/year	$/year	-	$16,000	$285,714	$200,000	$16,667
Annual labor cost	$/year	-	$5,000	$80,000	$30,000	$62,500
Other media or consumables	-	Milling media	Mixing blades	Molds	Kiln furniture	Fittingsetc.
Total media cost	$/year	$1,361	$13,609	$31,995	$117,742	$8,213
Total energy cost	$/year	$95	$95	$100	$6,181	$80
Disposal cost	$/(kg or tube)	$0.10	$0.10	$0.10	$0.10	$0.10
Material to dispose	(kg or parts)/year	0	2,722	5,119	4,121	5,475
Total disposal cost	$/year	-	$130.34	$470.98	$379.13	$503.70
Total cost	$/year	$1,456	$39,976	$478,321	$384,336	$125,507

**FIGURE 10:**
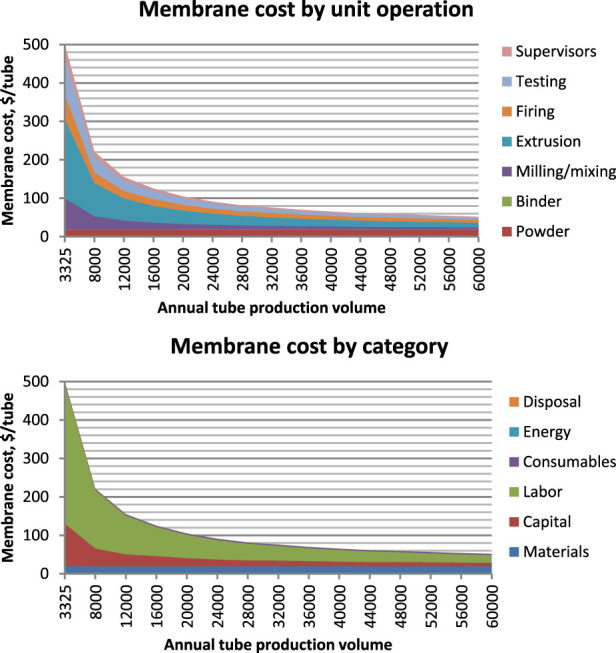
YSZ SOM cost vs. production scale broken down by unit operation (above) and cost category (below).

Figure 10 shows these costs graphically as a function of production scale, by unit operation and by cost category. YSZ purchase price also varies by production scale, but estimating this supply curve is beyond the scope of this study. Costs decline with increasing scale, leveling off around 30,000 tubes/year.

Compared with $2.95M/a cost for carbon anodes (Rutherford et al, 2021), or $147/t Mg, YSZ SOM anodes are more expensive by $51/t Mg, which is about 2.3% of the overall cost. Other effects on cost include.

• Higher energy consumption: at 1,050°C, the cell would operate at 8 V using YSZ SOM anodes vs. 5 V using carbon anodes, consuming 17.6 MWh/t vs. 11.0 MWh/t, due to the higher reduction potential for oxygen anodes and increased resistance with YSZ. At the reference case electricity price of $35/MWh, this adds about $230/t to Mg cost, i.e., about 10%.
• Higher cost of raw material: the bath would require Y₂O₃ addition in order to prevent yttria leaching from YSZ tubes, this adds 3% to bath cost or $2/t Mg.
• Lower cost of emissions control, and possible oxygen revenue: the plant would require much less ventilation and air handling, simplifying the plant design and operations, though estimating this cost savings is beyond the scope of this study. It would produce about 13 kt/a high purity oxygen gas, though monetizing this by-product stream would require a suitable local customer.

Note that 8V may seem too high for SOM electrolysis, as it is much higher than the reduction potential of both zirconia and yttria. This does not happen for two reasons. First, as shown in Figure 9, there are multiple overpotentials in the series which add up to this potential, including Mg reduction potential and ohmic overpotentials in the molten salt, anode and cathode leads, and YSZ itself. Second, reduction of YSZ requires an electronic pathway from the cathode, or a more electronegative cation available for reaction, and the molten salt electrolyte blocks electronic conduction from the cathode. In our experiments to date, we did not observe any blackening of the YSZ SOM tube, which would have indicated partial reduction.

### 4.3 Cradle-to-gate emissions estimation

**FIGURE 11:**
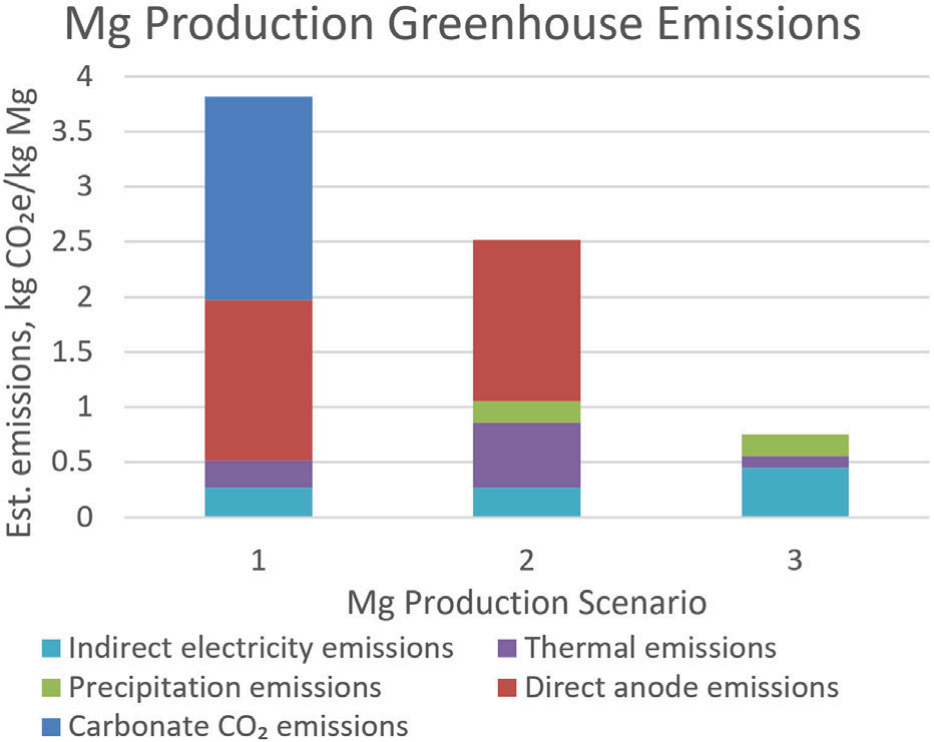
GHG emissions for Mg production in the high, medium, and low emission scenarios.

Figure 11 shows estimated emissions for the 3 Mg production scenarios described above. Direct emissions are due to carbon anode reactions and natural gas-fired drying and calcination in the high and medium emission scenarios. Indirect emissions are from generation of electricity used in electrolysis, distillation, and raw material production (for example, NaOH made by the chlor-alkali process). Carbonate CO₂ emissions are from decomposition of magnesite ore; use of dolomite would roughly double this value. In Figure 11: “Indirect electricity emissions” refers to those associated with electricity generation for electrolysis; “Thermal emissions” refers to those from gas burning or electricity generation for drying and calcining MgCO_3_ or Mg(OH)_2_; “Precipitation emissions” refers to electricity generation for the chlor-alkali process used to generate NaOH precipitation agent; “Direct anode emissions” refers to those from the carbon anode reaction described above; and “Carbonate CO_2_ emissions” refers to those from MgCO_3_ decomposition.

## 5 Discussion

As discussed above, Mg production operating cost using the above method is potentially low. The biggest energy and cost savings relative to conventional MgCl₂ electrolysis is in the much simpler and less energy-intensive drying of MgO, though the inability to use a multi-polar cell for MgO reduction raises the minimum electrolysis energy consumption.

Operating cost is sensitive to prices of electricity, MgO raw material, carbon or YSZ anodes, and tin for the reactive cathode. These experiments with carbon anodes have demonstrated current yield of 94%–97% which is comparable to 93%–95% in the aluminum industry, though it remains to be seen whether this persists over longer time and at scale.

It is possible that the current yield in the SOM anode experiment is lower than with carbon anodes due to the presence of Y³⁺ in the electrolyte. That is, although Y₂O₃ is slightly more stable than MgO, the preponderance of F⁻ ions in the electrolyte could make Mg more stable, as YF₃ is less stable than MgF₂, and yttrium is reduced. Y³⁺ in the electrolyte dramatically reduces yttria leaching from the SOM, so removing it would require using an alternative such as calcia-stabilized zirconia (CSZ) or magnesia-stabilized zirconia (MSZ) for the SOM instead of YSZ. With lower conductivity of CSZ or MSZ, it would be necessary to make the SOM thinner in order to avoid higher resistance and more energy consumption.

As mentioned, lead (Pb) is a potential alternative to tin for this process, with many advantages. At the time of this writing, Pb at $2,050/t is much less expensive than Sn at $26k/t. The higher density of Pb allows for more Mg absorption while maintaining higher density than the electrolyte. That higher starting Mg fraction, plus the higher activity coefficient for Mg in Pb than Sn, reduce the energy required for distillation. And Fe solubility in Pb is much lower than in Sn, so a nickel-free stainless material of construction for the distiller, such as alloy 440C, would not corrode as much with liquid Pb-Mg alloy. In production plant design, these advantages must be carefully weighed against the much more stringent handling protocols required for industrial use of Pb.

Regarding other potential reactive liquid metal cathodes.

• Cu and Ag melting points are much higher than those of Sn and Pb, making it much harder to remove electronegative raw material impurities such as Fe, Al and Si via temperature swing.
• Ag price at $740k/t is much higher than others, so small losses would dominate overall cost.
• The very low Mg activity coefficient in Sb makes distillation very energy-intensive.

Fundamentally, this process is very similar to Hall-Héroult aluminum (Al) production. Because Mg is divalent rather than trivalent, electrolysis produces 50% more Mg by mole and 33% more Mg by mass per A·h charge than Al, particularly if the 94%–97% current yield measured here holds up at scale. Cell voltage with carbon anodes is very similar to that of Al cells, so electrical energy consumption would be lower than for Al, and MgO raw material cost is also very close to that of alumina. At scale, this process could therefore produce Mg at equal or lower cost vs. Al. With the same carbon anodes, current bus, cell configuration and materials, raw material feed and product material withdrawal systems, and electrical infrastructure, this Mg production process could even retrofit into an existing or curtailed alumina smelter to dramatically reduce capital cost.

For production using carbon anodes, G-METS distillation is likely the biggest technical risk: a continuous distillation process would have low capital and labor costs as well as low energy usage, but has not been proven. If continuous distillation is not feasible, conventional distillation with 6–10 h cycle time and 5-7 kWh/kg energy usage would raise the cost of Mg-cathode separation considerably.

Using YSZ SOM anodes, the biggest cost increase vs. carbon anodes is for energy due to the high electrical resistance of the zirconia membrane, resulting in $230/t Mg product additional cost, vs. $51/t in higher costs for the anodes themselves, at 4,000-h lifetime. If anode lifetime is just 2,000 h, then anode cost is about $100/t, *etc.* If a low-cost support could replace much of the YSZ thickness without sacrificing lifetime, then reductions in cell voltage and energy cost, along with reduced zirconia powder use, could result in lower overall cost despite the increased anode manufacturing complexity.

Though a full LCA is not yet complete, emissions calculated for this process are relatively low. In particular, using hydroxide raw material from brines or seawater instead of carbonates, and substituting electrical energy for fossil fuel energy in raw material production, can dramatically reduce emissions. As renewable electrical energy cost continues to fall relative to fossil fuel energy, this can potentially make the lowest-emissions scenario also the one with lowest cost.

## 6 Conclusion

At 94%–97% with carbon anodes and 84% with YSZ SOM anodes, the current yields measured in this study are the highest for electrolytic MgO reduction in molten MgF₂-CaF₂ salt. This is likely due to the use of a reactive tin cathode which reduces the activity of the Mg product, which both increases the reduction reaction driving force, and also reduces metallic Mg dissolution and electronic conductivity in the electrolyte.

Costs with YSZ SOM oxygen-producing anodes are around 10%–15% higher than with carbon anodes. Based on the model assumption of 4,000-h membrane lifetime, and cost estimates of YSZ powder and SOM tube manufacturing at scale, cost of the YSZ membranes themselves only increases Mg production cost by about 2% relative to carbon anodes; the remainder of the cost increase is due to increased cell resistance resulting in higher electrical energy usage.

A large fraction of Mg production GHG emissions using this process is due to raw material production and carbon anode consumption. Using MgCl₂ from brines or seawater, electrical energy for NaOH precipitating agent, electrical heating energy, and YSZ SOM anodes can dramatically reduce process emissions.

## Data Availability

The raw data supporting the conclusion of this article will be made available by the authors, without undue reservation.
